# Doublet method for very fast autocoding

**DOI:** 10.1186/1472-6947-4-16

**Published:** 2004-09-15

**Authors:** Jules J Berman

**Affiliations:** 1 Cancer Diagnosis Program, National Cancer Institute, National Institutes of Health, Bethesda, MD, USA

## Abstract

**Background:**

Autocoding (or automatic concept indexing) occurs when a software program extracts terms contained within text and maps them to a standard list of concepts contained in a nomenclature. The purpose of autocoding is to provide a way of organizing large documents by the concepts represented in the text. Because textual data accumulates rapidly in biomedical institutions, the computational methods used to autocode text must be very fast. The purpose of this paper is to describe the doublet method, a new algorithm for very fast autocoding.

**Methods:**

An autocoder was written that transforms plain-text into intercalated word doublets (e.g. "The ciliary body produces aqueous humor" becomes "The ciliary, ciliary body, body produces, produces aqueous, aqueous humor"). Each doublet is checked against an index of doublets extracted from a standard nomenclature. Matching doublets are assigned a numeric code specific for each doublet found in the nomenclature. Text doublets that do not match the index of doublets extracted from the nomenclature are not part of valid nomenclature terms. Runs of matching doublets from text are concatenated and matched against nomenclature terms (also represented as runs of doublets).

**Results:**

The doublet autocoder was compared for speed and performance against a previously published phrase autocoder. Both autocoders are Perl scripts, and both autocoders used an identical text (a 170+ Megabyte collection of abstracts collected through a PubMed search) and the same nomenclature (neocl.xml, containing over 102,271 unique names of neoplasms). In side-by-side comparison on the same computer, the doublet method autocoder was 8.4 times faster than the phrase autocoder (211 seconds versus 1,776 seconds). The doublet method codes 0.8 Megabytes of text per second on a desktop computer with a 1.6 GHz processor. In addition, the doublet autocoder successfully matched terms that were missed by the phrase autocoder, while the phrase autocoder found no terms that were missed by the doublet autocoder.

**Conclusions:**

The doublet method of autocoding is a novel algorithm for rapid text autocoding. The method will work with any nomenclature and will parse any ascii plain-text. An implementation of the algorithm in Perl is provided with this article. The algorithm, the Perl implementation, the neoplasm nomenclature, and Perl itself, are all open source materials.

## Background

Autocoding is a specialized form of machine translation. The general idea behind machine translation is that computers have the patience, stamina and speed to quickly parse through gigabytes of text, matching text terms with equivalent terms from an external vocabulary. Human translators often scoff at the output of machine translators, noting the high rate of comical errors. An often cited, perhaps apocryphal, example of poor machine translation is the English to Russian transformation of "out of sight, out of mind" to the Russian equivalent of "invisible idiot."

Despite limitations, machine translation is the only way to transform gigabytes and terabytes of text. As long as clinicians, pathologists, radiologists, nurses, and scientists continue to type messages, reports, manuscripts and notes into electronic documents, we will need computers to parse and organize the resulting text.

One of the many problems in the field of machine translation is that expressions (multi-word terms) convey ideas that transcend the meanings of the individual words in the expression. Consider the following sentence:

"The ciliary body produces aqueous humor."

The example sentence has unambiguous meaning to anatomists, but each word in the sentence can have many different meanings. "Ciliary" is a common medical word, and usually refers to the action of cilia. Cilia are found throughout the respiratory and GI tract and have an important role locomoting particulate matter. The word "body" almost always refers to the human body. The term "ciliary body" should (but does not) refer to the action of cilia that move human bodies from place to place. The word "aqueous" always refers to water. Humor relates to something being funny. The term "aqueous humor" should (but does not) relate to something that is funny by virtue of its use of water (as in squirting someone in the face with a trick flower). Actually, "ciliary body" and "aqueous humor" are each examples of medical doublets whose meanings are specific and contextually constant (i.e. always mean one thing). Furthermore, the meanings of the doublets cannot be reliably determined from the individual words that constitute the doublet, because the individual words have several different meanings. Basically, you either know the correct meaning of the doublet, or you don't.

Any sentence can be examined by parsing it into an array of intercalated doublets:

"The ciliary, ciliary body, body produces, produces aqueous, aqueous humor."

The important concepts in the sentence are contained in two doublets (ciliary body and aqueous humor). A nomenclature containing these doublets would allow us to extract and index these two medical concepts. A nomenclature consisting of single words might miss the contextual meaning of the doublets.

What if the term were larger than a doublet? Consider the tumor "orbital alveolar rhabdomyosarcoma." The individual words can be misleading. This orbital tumor is not from outer space, and the alveolar tumor is not from the lung. The 3-word term describes a sarcoma arising from the orbit of the eye that has a morphology characterized by tiny spaces of a size and shape as may occur in glands (alveoli). The term "orbital alveolar rhabdomyosarcoma" can be parsed as "orbital alveolar, alveolar rhabdomyosarcoma" Why is this any better than parsing the term into individual words, as in "orbital, alveolar, rhabdomyosarcoma"? The doublets, unlike the single words, are highly specific terms that are unlikely to occur in association with more than a few specific concepts.

Very few medical terms are single words. In "The developmental lineage classification and taxonomy of neoplasms" there are 102,271 unique terms for neoplasms. All but 252 of these terms are multi-word terms [see Additional file 1] [see Additional file 2][1]. Of the 252 singletons, all but 34 are names of specific tumors ending in the suffix, "oma." "Oma" is short for "tumor." Single-word names of tumors ending in "oma" can be thought of as doublets with the first and second words fused together (i.e. osteoblastoma is "osteoblast" + "oma"). Some examples of "oma" terms are: acanthoma, adamantinoma, adenofibroma, adenomyoepithelioma, adenomyoma, adenosarcoma, ameloblastoma, etc.) In the entire taxonomy, there are only 34 singletons that do not end in "oma." These are, "acrochordon, carcinoid, cyst, dermoid, dip-nech, dipnech, erythroleukemia, fibroid, histiocytosis, leucaemia, leukaemia, leukemia, macroglobulinemia, mastocytosis, milia, milium, myelodysplasia, naevus, neuronevus, nevus, parapsoriasis, pre-leukaemia, pre-leukemia, precancer, preleukemia, premalignancy, preneoplasia, tylosis, verruca, verrucae, and wart". These singletons represent a mere 34/102,271 or 0.0003 of the neoplasm terminology.

Medical autocoding can be considered a specialized form of machine translation. Medical autocoders transform text into an index of coded nomenclature terms (sometimes called a "concept index" or "concept signature"). Several innovative approaches to autocoding have used the higher information content of multiword terms (also called word n-grams) to match terms in text with terms in vocabularies or to enhance the content of vocabularies by identifying n-grams occurring in text that qualify as new nomenclature terms [2-4]. Unlike prior studies with n-grams, the method developed for this study does not use statistical inferencing or the information content of bi-grams to infer semantic meaning from natural language. The author has used the higher term specificity of doublets [bi-grams] to construct a simple and fast lexical parser. Lexical parsers are types of string-matching algorithms. In general, the overall speed of lexical parsers is determined by the speed with which the parser can prepare an array of all possible words and phrases contained in a block of text, coupled with the speed with which each of these phrases can be compared against all the terms in the nomenclature.

The purpose of this paper is to describe a novel algorithm for autocoding based on finding runs of word-doublets that match a list of doublets extracted from a medical nomenclature. Using the same hardware and the same nomenclature, the speed and accuracy of the doublet method can be compared with another fast lexical parser.

## Implementation

### Nomenclature

The nomenclature used is neocl.xml, previously described by the author and currently designated as "The developmental lineage classification and taxonomy of neoplasms"[1]. In the context of this manuscript, the purpose of the taxonomy is to provide a listing of all names of neoplasms, with synonyms grouped under a common code number. The current version of the neocl.xml file contains 102,271 unique names of neoplasms. In constructing the taxonomy, enormous effort was made to include every variant name for every known neoplasm of man. Variant names included different terms for the same concept and different ways of expressing an individual term (e.g. variations in word order).

An example of the variety of synonyms encountered for a single tumor is shown for "adenocarcinoma of the colon." There are 44 synonyms listed in the taxonomy. These are: adenoca arising from colon, adenoca arising in colon, adenoca of colon, adenocarcinoma arising from colon, adenocarcinoma arising from large intestine, adenocarcinoma arising from the colon, adenocarcinoma arising from the large intestine, adenocarcinoma arising in colon, adenocarcinoma arising in large intestine, adenocarcinoma arising in the colon, adenocarcinoma arising in the large intestine, adenocarcinoma of colon, adenocarcinoma of large intestine, ca arising from colon, ca arising in colon, ca of colon, cancer arising from colon, cancer arising in colon, cancer of colon, carcinoma arising from colon, carcinoma arising in colon, carcinoma of colon, colon adenoca, colon adenocarcinoma, colon ca, colon cancer, colon cancers, colon carcinoma, colon carcinomas, colon with adenoca, colon with adenocarcinoma, colon with ca, colon with cancer, colon with carcinoma, colonic adenoca, colonic adenocarcinoma, colonic adenocarcinomas, colonic ca, colonic cancer, colonic cancers, colonic carcinoma, colonic carcinomas, large intestine adenocarcinoma, large intestine with adenocarcinoma.

### Input file

The input file was created by a PubMed query on "pathology [ad] AND neoplasm [all]", at the U.S. government website [5]. The query gathered all abstracts from the pubmed database in which the term neoplasm occurs somewhere in the pubmed entry, and in which the affiliation of the author contains the word "pathology". The query yielded abstracts that are likely to contain names of neoplasms. The PubMed output file can serve as a good test for an autocoder that uses a neoplasm nomenclature. The PubMed search yielded 66,509 abstracts. All of the abstracts were downloaded into a single file from the PubMed site by setting the "Display" attribute to "Medline" and the "Send to" attribute to "file". This produced a 170,997,880 byte plain-text file. The file was given the filename tumor.txt, and this filename was used by the autocoders as a parsing input file. Although this file is not included with this manuscript, anyone in the world with internet access can obtain a near-identical file by repeating the same PubMed query.

### Doublet autocoder

The doublet autocoder is supplied as an open source Perl script (doubcode.pl) with this manuscript [see Additional file 3]. Perl itself is an open source language that is bundled with the Unix and Linux operating systems. In the past decade, Perl has become very popular in the bioinformatics community. Perl interpreters are available at no cost and in versions suitable for virtually all operating systems. Perl can be obtained from the Comprehensive Perl Archives Network [6] or from Active State [7].

The algorithm used by the doublet autocoder is described:

1. Each phrase (term) in the nomenclature (neocl.xml) is converted into intercalated doublets, and each doublet is assigned a consecutive number.

2. Each nomenclature phrase is assigned the concatenated list of numbers that represent the ordered doublets composing the phrase.

3. Every text record (pubmed abstract in this case) is split into an array consisting of the consecutive words in the text record.

4. The text array is parsed as intercalated doublets. Intercalated doublets from the text that match doublets found anywhere in the nomenclature are assigned their numeric values (from the doublet index created for the nomenclature). Runs of consecutive doublets from the text that match doublets from the nomenclature are built into concatenated strings of doublet values. The occurrence of a text doublet that does not match any doublet in the nomenclature cannot possibly be part of a nomenclature term. Such text doublets serve as "stop" doublets between candidate runs of text doublets that match nomenclature doublets.

5. The runs of matching doublets are tested to see if they match any of the runs of doublets that compose nomenclature terms or if they contain any subsumed terms that match nomenclature terms.

6. The array of doublet runs extracted from the text that match nomenclature terms are cached in an external file.

### Phrase autocoder

The phrase autocoding script, phrase.pl is included as supplementary file with this article [see Additional file 4]. The script was described in a prior publication [8]. It works by taking text records and parsing the text into all possible ordered phrases of all sizes up to a predetermined limit (5, in this case). The script then examines each resultant phrase to determine if it matches a term in the nomenclature. If there is a match, the term is added to the cache of matched terms.

## Results

### Speed

The speed of both coders was compared using a 170+ Megabyte text. The doublet method coded the entire text in 211 seconds compared with 1,766 seconds required for the phrase method. Therefore the doublet method coded 8.4 times faster than the phrase method. The text coding rate for the doublet method is 0.8 Megabytes per second on a desktop computer with a modest 1.6 GHz Pentium processor.

Both the phrase method and the doublet method coders used the same Programming Language, the same nomenclature, and the same data structure for the nomenclature (a simple associative array). Both coders grab sequentially occurring chunks of text, each chunk delimited by a double-newline delimiter (roughly the ascii equivalent of a paragraph delimiter) and place the grabbed text chunk into a temporary scalar variables.

The difference between the two coders occurs when the chunks of text are parsed. The phrase coder takes the text and creates an array of every ordered combination of 1,2,3,4 and 5-word phrases contained in the record. This number of elements in the phrase array is about 5 times larger than the number of words in the record. The doublet coder parses the record into the set of all ordered doublet terms. This number of elements in the array of doublets is about the same as the number of words in the record. The phrase coder must try to match about 5 times as many terms as the doublet coder. Also, the phrase coder tries to match each phrase in the record array against the entire nomenclature. The doublet coder tries to match each doublet encountered in the input text against the collection of doublet terms extracted from the nomenclature. Because doublets of text, unlike individual words, tend to have unique meanings, only a small subset of the doublets encountered in the input text will match the set of doublets extracted from the nomenclature. Text doublets that do not match any doublets in the nomenclature are "skipped." Text doublets that match doublets from the nomenclature are concatenated to consecutive matching doublets until a non-matching doublet is encountered. The length of matching doublets sets the length of the candidate term. The algorithmic strength of the doublet method is that it eliminates the need to create and match [against a nomenclature] an array of all possible phrases of all possible lengths found in a textual record.

### Autocoding output

The output of the doublet method and phrase method autocoders are provided as supplemental files with this manuscript [see Additional file 5] [see Additional file 6]. The analysis of the autocoding output was performed using another Perl script, doubcode.pl [see Additional file 7]. The analysis output file is also provided with this manuscript [see Additional file 8].

The two autocoder methods parsed a text exceeding 170 megabytes in length. The doublet method coded 4807 different [sometimes called unique] terms. The total number of matching term encountered (which includes replicate matches of terms within a record) by the doublet method is 467,391 terms. The phrase method coded 4557 different terms. There were 250 terms that the doublet method matched and the phrase method missed. There were zero terms that the phrase method matched and the doublet method missed.

The improved performance of the doublet coder is accounted for by its ability to match terms of any length, while the phrase coder is limited to match terms that are no more than 5 words in length. Because the phrase coder parses chunks of input text into all allowed phrase size combinations, the performance of the phrase coder is slowed dramatically when the allowed phrase length is large. For instance, if the theoretical maximum phrase size were permitted for a text chunk 100 words in length, the phrase parser would need to create an array of a size equal to 100 × 100 or 10,000 phrases, matching each phrase against nomenclature. If the phrase size were restricted to 5 words, then the phrase parser would only need to create a phrase array of size 5 × 100 or 500. It is reasonable to restrict the allowed length of coded term phrases if it is known that the number of nomenclature terms with length exceeding the allowed limit is small. It is also reasonable to restrict the allowed length of coded term phrases is it is believed that terms of long length can be successfully represented by subsumed terms within the longer term.

For example, "Refractory anemia with excess blasts in transformation" will be captured as a single term by the doublet method. The phrase method will miss the 7-word term but will code the subsumed terms, "refractory anemia" and "refractory anemia with excess blasts". In this specific instance, the doublet coder outperformed the phrase coder. However, few cancer terms actually exceed 5 words in length, and longer terms almost always contain modifying phrases (so-called term pre-coordinations) that add little to the fundamental meaning of the term.

The following terms are examples of neoplasms extracted by the doublet method and missed by the phrase method. All of the listed terms exceed 5 words in length. "Acute myelogenous leukemia with minimal differentiation, acute myeloid leukemia with minimal differentiation, adenocarcinoma arising in the parotid gland, adenoid cystic carcinoma of submandibular gland, aids-related primary central nervous system lymphoma, atypical glandular cells of undetermined significance, atypical squamous cells of uncertain significance, carcinoid tumor arising from meckel diverticulum, extranodal marginal zone b-cell lymphoma of mucosa-associated lymphoid tissue, giant cell tumor of tendon sheath, giant cell tumour of tendon sheath, glandular malignant peripheral nerve sheath tumor, high-grade malignant peripheral nerve sheath tumor, hurthle cell carcinoma arising from thyroid, hyalinizing spindle cell tumor with giant rosettes, interdigitating dendritic cell sarcoma arising from the spleen, intermediate-grade b-cell non-hodgkin lymphoma, intraductal papillary mucinous neoplasm of pancreas, intraductal papillary-mucinous tumor with moderate dysplasia, kaposi sarcoma arising in the heart, large cell calcifying sertoli cell tumor, leiomyosarcoma arising from the pulmonary artery, low grade cervical glandular intraepithelial neoplasia, low-grade malignant peripheral nerve sheath tumor, lymphoma arising in the thyroid gland, malignant epithelioid peripheral nerve sheath tumor, malignant tumor of peripheral nervous system, malt lymphoma arising in the stomach, metanephric adenosarcoma in a young adult, monomorphic clear cell adenocarcinoma of salivary glands, ovarian serous borderline tumor with micropapillary and cribriform patterns, ovarian serous tumor of low malignant potential, paraganglioma arising in the cauda equina, perineurial malignant peripheral nerve sheath tumor, philadelphia chromosome negative chronic myeloid leukemia, phosphaturic mesenchymal tumor mixed connective tissue variant, pineal parenchymal tumor of intermediate differentiation, pleomorphic adenoma of minor salivary glands, polymorphous low-grade adenocarcinoma of minor salivary gland, polymorphous low-grade adenocarcinoma of minor salivary glands, polymorphous low-grade adenocarcinoma of salivary gland, poorly differentiated malignant peripheral nerve sheath tumor, porokeratotic eccrine ostial and dermal duct nevus, primary cutaneous anaplastic large cell lymphoma, primary gastric diffuse large b-cell lymphoma, primary signet-ring cell carcinoma of lung, primitive neuroectodermal tumor arising in the pancreas, pulmonary immunocytoma with massive crystal storing histiocytosis, rhabdoid transformation of tumor cells in meningioma, serous ovarian tumor of low malignant potential, sex cord tumor with annular tubules, signet ring cell adenocarcinoma of prostate, signet-ring cell mucin-producing adenocarcinoma of minor salivary glands, sinonasal desmoplastic small round cell tumor, smooth muscle tumor of uncertain malignant potential, smooth muscle tumor with an uncertain malignant potential, splenic lymphoma with circulating villous lymphocytes, squamous cell carcinoma of fallopian tube, submandibular gland carcinoma ex pleomorphic adenoma, tall cell variant of papillary carcinoma of thyroid, testicular large cell calcifying sertoli cell tumor, uterine tumor resembling ovarian sex cord tumor."

The long terms found by the doublet coder and missed by the phrase coder represent a very small percentage of the different terms found by either coder in the long 170+ Megabyte text (250/4807 = 5%). A cursory review of these terms indicates that they represent rare lesions or rare variants of common lesions.

## Discussion

Developers of medical autocoders seldom publish manuscripts. It is the author's perception, based on many years of activity in this field, that most autocoders are proprietary products produced for a very specific type of job. The author has never encountered any autocoder vendors who revealed the speed of their autocoders or shared any primary data that measures the performance of their autocoders. There really has never been any publication where one autocoder was compared against another. This is unfortunate because software developers may defer implementing brilliant ideas for autocoders, if they are uncertain whether better autocoders already exist. Even if competing software developers were to share performance data, there are no widely accepted standards for measuring the performance of medical autocoders. One of the purposes of this paper is to provide open access to algorithms, software, performance methodology and performance outcome data.

### Accuracy issues for lexical parsers

The performance of autocoders is always a contentious issue. The reflexive approach to measuring autocoder performance usually involves selecting a small corpus of text and allowing a human expert in a language domain to carefully read through the lines of the text, creating a so-called gold standard against which the autocoder can be compared. The problems with this seemingly straightforward approach are many and have been explained by myself and others. The many limitations of "precision and recall" as measurements of indexing performance have been reviewed elsewhere [9,10].

It would be a useful exercise to re-examine what autocoding performance means to different people in contrast to what autocoding performance means within the context of lexical parsers [11,12]. First, every human coder is biased by their different perceptions of the knowledge domain. One coder may prefer "parsimonious" coding. In "parsimonious" coding, there is a "best" code that represents the ideas contained in a defined section of text. A review article on "liver cirrhosis" may contain many different terms, but the parsimonious coder may only preserve a single code for the entire article. Another coder, also a parsimonious coder, may not be so strict, but may want only the best term from among a group of subterms. So if "adenocarcinoma of endometrium" appears in text, she may want to preserve this term but omit the so-called atomic inclusive terms, "adenocarcinoma" and "endometrium." Another coder may want a complete listing of every matching term in a text, including terms that occur within larger terms. Still another informatician may want to include all ancestral terms for each term found in the text (i.e. terms not present in the original text but related to the textual concept).

Finally, some coders seek to create "concept signatures" from text. A concept signature is the list of the concepts contained in a section of text. The relationship of one section of text to another section of text is determined by a quantitative representation of how closely their signatures match. Concept signatures are used to retrieve or organize related documents, not specific concepts [13].

Lexical coders are, in a sense, perfect autocoders because they don't use grammatic rules, they have no "exception" lists, and they never guess or interpret text. The only thing that lexical parsers do is to parse text, examining strings to see if they contain exact matches to terms from a nomenclature. When well-constructed, they do not make mistakes. If they miss a valid term that is present in the text, it is because the term was missing from the nomenclature. Developers of lexical autocoders place enormous demands on the curators of nomenclatures.

The most common cause of missed terms arises when the text contains a modifying word that breaks the term into a phrase that no longer matches anything found in the nomenclature. For instance, "adenocarcinoma of the left lung," would be missed by a lexical parser if the nearest term in its terminology list is "adenocarcinoma of the lung." A rule-based parser or a semantic parser may have successfully teased out the "left" from the term and found the match. In this instance, the nomenclature failed the lexical parser because it did not list terms that indicated tumor laterality. Lexical parsers do not match terms that are absent from the text (i.e. no false positives). False-positive terms are possible in rule-based and semantic parsers if they create word patterns not present in the original text. Also, since lexical parsers strictly match phrases in text with phrases in nomenclatures, it is possible to achieve accurate results of dubious value. For instance, a lexical parser would parse "adenocarcinoma of the lung is not seen" and find a match against the neoplasm term, "adenocarcinoma of the lung." The concept is present in the text, even if though it is absent from the patient. It is a misleading but "true" positive.

The doublet autocoder creates a string of words from a chunk of text. In the case of the example corpus (PubMed abstracts), all of the sentences from the abstract are squeezed into a single string of words, obliterating sentence boundaries. This means that if a sequence of words crossing a sentence boundary happens to match a term in the reference nomenclature, the coder will register a "pseudo-positive" term. Empirical evidence suggests that this theoretical error in the doublet autocoder simply does not occur. A 4 megabyte collection of abstracts chunked as whole abstracts or as chunked sentences (delimited by period-space-space) contained no instances of pseudo-terms created by the obliteration of sentence boundaries in the whole-abstract text chunks. For fastidious developers who wish to ensure that their parsers respect sentence boundaries, it is possible to pre-process text into sentences with a sentence parser [14,15].

Lexical parsers can be modified to provide an output that preserves the intended sense of the term as used in its context. For example, if the text includes the sentence, "Adenocarcinoma of the lung is not present." The "sensible" lexical parser may be modified to tag the "adenocarcinoma of the lung" with a negation modifier, preserving the intended sense of the term. The author has previously published an "in place" method of inserting codes directly into sentences, preserving modifier terms (including negations) [16].

### Comparing parsers

The two lexical parsers used the same programming language (Perl), the same nomenclature, the same data structure to hold the nomenclature, the same text corpus, and the same method for breaking the complete text into text chunks. Differences between the performance of the two autocoding methods were accounted for entirely by the algorithmic processes by which chunks of text are parsed into phrases that are matched against the common nomenclature. It is easy to compare speeds. The doublet parser required 211 seconds to parse the 170+ Megabyte file, while the phrase parser required 1,776 seconds. Three algorithmic properties contribute to the speed advantage of the doublet coder over the phrase coder: 1) The doublet coder parses text into a number of phrases that is 5-fold smaller than the number of phrases produced by the phrase coder; 2) Doublets that are not found in any of the nomenclature terms can be quickly excluded, 3) Successive text doublets that match doublets from the nomenclature can be quickly concatenated and tested for matches in the nomenclature. These are the algorithmic differences between the phrase coder and the doublet coder and presumably account for the improved speed of the doublet method.

Comparing coding accuracy is somewhat trickier. As discussed, lexical parsers have no "false positive" matches. Lexical parsers, unlike natural language processors, do not construct context-based new word phrases from native text. Lexical parsers only match exact phrases found in text with exact nomenclature terms. Therefore, neither the doublet coder nor the phrase coder have false-positive term matches. Coding output was compared by examining the different terms matched by the two coders. The number of different terms found by the doublet method was 4,807, compared with 4,557 terms found by the phrase method. There were no terms found by the phrase method and missed by the doublet method. There were 250 terms missed by the phrase method and found by the doublet method (about 5% of the number of different terms found by the doublet method). For the most part, these terms represented rare neoplasms with pre-coordinated modifiers. The reason that such rare terms were encountered is due to the large size of the text (exceeding 170 Megabytes). When the doublet and phrase methods are judged by their ability to extract nomenclature from text, their output would be similar for small text sizes or when the primary purpose of the coding is to extract and count commonly occurring terms.

## Conclusions

The doublet method is a novel approach to autocoding. It can autocode 0.8 Megabytes of text per second on desktop computer using a modest 1.6 GHz processor. For this manuscript, the doublet autocoder was tested on a corpus of medical text that exceeded 170 Megabytes in length and used a publicly available nomenclature of neoplasia containing 102,271 unique terms. In a side-by-side comparison with a publicly available fast lexical autocoder, the doublet method was 8.4-fold faster. The doublet algorithm is generalizable to any type or length of plain-text using any nomenclature. The algorithm and the Perl implementation script are available as open source documents.

## Availability and requirements

The autocoding scripts are short programs written in Perl. Perl is a freely available open source programming language. Perl interpreters for virtually any operating system are available from several sites on the web [6,7]. These sites have links to rich sources of online information on the Perl language. The autocoding script will execute on any operating system hosting a Perl interpreter. It requires an external plain-text file (to be autocoded) and a nomenclature. With minor modification, the Perl scripts will use any parsable nomenclature that contains listed plain-text terms associated with alphnumeric concept codes.

The Perl scripts (doublet.pl and phrase.pl) will work without modification using the current version of "The developmental lineage classification and taxonomy of neoplasms," which is provided with this manuscript [see Additional file 1] [see Additional file 2].

## Competing interests

None declared.

## Authors' contribution

The work expresses the opinion of the author and does not represent policy of the U.S. government.

## Pre-publication history

The pre-publication history for this paper can be accessed here:



## Supplementary Material

Additional File 1 Neocl.xml is the developmental lineage classification and taxonomy of neoplasms, in XML format. Because neocl.xml exceeds 7 Megabytes when uncompressed, a gzipped version of the file is provided (neoclxml.gz). After downloading from the biomedcentral site, the filename should be provided with a .gz suffix (if absent from the filename as downloaded). After decompressing the file, the file shoud be renamed "neocl.xml". The file can be viewed on current web browsers, but experience has shown that many browsers lack sufficient memory to display the entire file. Otherwise, the file can be viewed on a wordprocessor or an ascii editor. Click here for file

Additional File 2 Neoself.gz is the ascii flat-file version of the neocl.xml file. The file exceeds 16 Megabytes when expanded. Each line-record of the file consists of the numbered name of a different term, its code value, and the hierarchical list of its ancestral [class] terms from the developmental lineage classification and taxonomy of neoplasms. Click here for file

Additional File 3 Doubcode.pl is a Perl script that implements the doublet method for text autocoding and expects an external file named tumor.txt as the source corpus and a file named neocl.xml as the reference nomenclature. Click here for file

Additional File 4 Phrase.pl is a Perl script that implements the doublet method for text autocoding and expects an external file named tumor.txt as the source corpus and a file named neocl.xml as the reference nomenclature. Click here for file

Additional File 5 Doub.out is the plain-text output of the doublet method for text autocoding (i.e. output of doubcode.pl). Click here for file

Additional File 6 Quickcan.txt is the plain-text output file for phrase method for text autocoding (i.e. output of phrase.pl). Click here for file

Additional File 7 Doubcomp.pl is the Perl script comparing the output files produced by doubcode.pl and phrase.pl. Click here for file

Additional File 8 Doubcomp.txt is the plain-text output of doubcomp.pl. Click here for file
